# Clinical Instruction in the Hospital of Vienna

**Published:** 1862-12

**Authors:** E. L. Holmes

**Affiliations:** Chicago


### CLINICAL INSTRUCTION IN THE HOSPITAL OF VIENNA.

**By E. L. HOLMES, M. D., of Chicago.**

I intended to give a description of the Clinics in the Hospital at Vienna before leaving that city. I was unable to do this, and now take the first opportunity since my return to Chicago of fulfilling my promise.

Students from the United States, who visit the European Hospitals, are usually most interested in the clinical lectures, and probably such lectures are of more practical importance than any others. I have stated some of the reasons why I consider the Clinical Instruction of Vienna to be superior to that of other cities for those students who wish to fit themselves for the practice of *general medicine and surgery*. Any one who intended to pursue a specialty would undoubtedly do well to visit the clinics of every large city. The same is also true of general medicine, when the student has ample means and sufficient time, with a knowledge of different languages. In other cities, celebrated for their clinical lectures, there are the following inconveniencies, which I have learned by my own experience and that of others: The clinics in all the hospitals and in the wards of the same hospital are given at the same hour of the day—usually between eight o'clock and ten in the morning. Little effort seems to me to be made to give the student full advantage of the valuable material collected in these hospitals. This is too much the case in many of our American hospitals. It is difficult to see why, in large cities with fine hospitals, the clinics of each hospital and of each ward should not be given, as far as possible, at different hours of the day, that the student may have the opportunity of hearing as many clinical lectures as he may desire.

In this regard the Vienna hospital is far superior. Early in the morning are two clinics in general medicine, followed by those of surgery. After these, at separate hours, are the lectures at the bed side on diseases of the eye, skin, syphilis, obstetrics, and in the "dead house" on pathology.

In addition to the public clinics, the student finds every facility for taking private instruction in the diagnosis, treatment and pathology of the diseases of nearly every organ in the system. In this way the student is enabled to gain the greatest amount of practical knowledge in the shortest time. The lectures themselves compare very favorably with those of any hospital in the world.

One of the most populbr clinical lecturers in Vienna is Prof. Oppolzer. One can scarcely conceive a more practical plan of imparting medical knowledge than that adopted by this distinguished lecturer. Gifted with great fluency of speech and possessing a wonderful degree of erudition in everything pertaining to the past and present history of medical literature, united with an immense experience in the observation and treatment of disease and in public instruction, he is able to make his lectures interesting and of the greatest good to his listeners. Not only students, but old practitioners testify by their continued presence to the great merit of these lectures.

Every patient, as soon as he enters the wards of the lecturer, is assigned to the care of a student, whose duty it is to make a careful examination of the symptoms and keep a record of the case as long as it remains in the hospital; at the clinic the professor questions the student in presence of the class in everything pertaining to the case, calling the attention of all to every important point and comparing it with other similar cases in the ward. At the same time the secretions are carefully examined by means of the microscope and test tube.

The clinics are usually about an hour and a half long and are given six days in the week.

The clinics of Prof. Scoda are also worthy of notice. Although he is much less fluent and generally considered less interesting in his manner than Prof. Oppolzer, his lectures are none the less instructive. They are principally upon diseases of the chest. There are a sufficient number of patients and ample opportunity for every student to examine each patient for himself. Private courses of instruction in auscultation and percussion are given by Professor Scoda's assistant.

These clinics are followed by those of Professors Schuh and Dumreicher in the surgical wards. The general plan of instruction is the same as above described. Everything relating to operations, to the diagnosis and treatment of injuries and surgical diseases is carefully taught, with cases enough to illustrate every important point. I should infer, after considerable observation, that injuries requiring surgical treatment, especially fractures, were rare in Vienna as compared with our own large cities.

The clinical lectures in obstetrics are particularly important to the American student, who has little opportunity of receiving clinical instruction in this branch of his profession in America.

There are upon the average, I think, eight births a day in the hospital. The patients are delivered in a room assigned for the purpose, and then carried to the wards, where they remain nine days, or until they are able to leave. The class is carefully instructed in the mode of making examinations per vaginam, and of learning the position of the fœtal head and body during labor. The whole process of parturition is thus learned by repeated observation. There are also private courses on the use of instruments and "turning," the cadaver, from which the viscera have been removed, serving the purpose of a manikin. A dead fœtus is placed in different positions in the pelvls, which the students are to examine in turn and give their diagnosis. After this the operation of turning or the application of forceps is made and the fœtus delivered. When the student has taken this course on the application of instruments, he is permitted to use them when necessary on the living subject in the lying-in wards. One thus has an opportunity of watching several hundred cases of labor, of having a small number under his own care and of learning practically the use of instruments; he is also taught the duties of the lying-in room in reference to the mother and infant during the nine days subsequent to delivery.

The clinics of Prof. Hebra on diseases of the skin are one of the most popular courses in the hospital. In his wards are nearly two hundred and fifty patients, and with this large number of cases he is able to illustrate all the important points in the commencement and progress of every form of skin disease. The wards are open to the students, but Prof. Hebra usually delivers his lectures in a small amphitheatre, the patients being brought before the class. After he has called the attention of the class to the points worthy of notice in each case, the patient passes from one patient to another, thus enabling each to examine him more closely. The male patients are wholly naked at the clinics; the females being dressed in loose garments, to permit a ready examination of any part of the body. These clinics are given five days in the week.

The lectures of Prof. Sigmund on syphilis are very popular. There are two or three hundred cases in his wards. A careful examination of these, in connection with the lectures of the Professor, will make the student more familiar with this disease than he can be after years of reading and observation in private practice.

Students interested in diseases of the eye will find the clinics of Professors Arlt and Jaeger interesting and instructive. In these wards are about two hundred patients. The student has ample opportunity of acquiring a knowledge of the use of the ophthalmoscope, of witnessing a large number of operations and of acquiring skill in the diagnosis of opthalmic disease.

In addition to the ordinary clinics as above described there are private courses of instruction in the clinical study of every class of disease, including treatment, operative and medical. These courses are scarcely less beneficial to the student than the others, as they give him an opportunity of reviewing what he has alreany learned from the different Professors.

I cannot close this short notice of the Clinics of Vienna without alluding to the facilities given the student for the study of pathology. There are upon the average five post-mortem examinations a day, at which students can be present, notice always being given when a patient dies, of the hour when the autopsy will be made. Generally, however, the students prefer to be present at the lectures of Prof. Rokitansky, at which all the morbid specimens of diseased organsf collected each day, are exhibited. The private course o

Rokitansky's assistant is very useful, since he not only demonstrates all the fresh specimens, but visits the great Pathological Museum, for which Rokitansky has so long labored, and explains all the preparations, illustrating the effects of the disease of each organ.

The Pathological Museum may justly be regarded with pride by the Medical School of Vienna. The building is a large fire-proof structure of stone and brick, erected at a cost, as I was informed, of $40,000. The Museum is a large hall, tastefully fitted and ornamented. The other portions of the building are used for the reception of the dead previous to burial, for dissections, for the ordinary post-mortem examination of the hospital, and for the examination of cases of sudden or violent death in the city. Each Professor has a private room for the examination of his own cases. Whatever may be said of the advantages offered by other cities for the study of surgery or medicine, I think no city can claim for its hospitals better facilities for the study of pathology than can be found in Vienna.

I trust I have been understood in what I have said regarding the superiority of the Medical School of Vienna. I have given my reasons based not only on my own experience, but especially on that of many other medical men from nearly every country of the world who have visited every large city of Europe.

